# Emerging Tuberculosis Pathogen Hijacks Social Communication Behavior in the Group-Living Banded Mongoose (*Mungos mungo*)

**DOI:** 10.1128/mBio.00281-16

**Published:** 2016-05-10

**Authors:** Kathleen A. Alexander, Claire E. Sanderson, Michelle H. Larsen, Suelee Robbe-Austerman, Mark C. Williams, Mitchell V. Palmer

**Affiliations:** a Department of Fish and Wildlife Conservation, Virginia Tech, Blacksburg, Virginia, USA; b CARACAL, Centre for Conservation of African Resources: Animals, Communities, and Land Use, Kasane, Botswana; c Department of Medicine, Albert Einstein College of Medicine, Bronx, New York, USA; d Diagnostic Bacteriology Laboratory, National Veterinary Services Laboratories, Ames, Iowa, USA; e University of Pretoria, Onderstepoort, South Africa; f Bacterial Diseases of Livestock Research Unit, National Animal Disease Center, Ames, Iowa, USA

## Abstract

An emerging *Mycobacterium tuberculosis* complex (MTC) pathogen, *M. mungi*, infects wild banded mongooses (*Mungos mungo*) in Northern Botswana, causing significant mortality. This MTC pathogen did not appear to be transmitted through a primary aerosol or oral route. We utilized histopathology, spoligotyping, mycobacterial interspersed repetitive units-variable number of tandem repeats (MIRU-VNTR), quantitative PCR (qPCR), and molecular markers (regions of difference [RDs] from various MTC members, including region of difference 1 [RD1] from *M. bovis* BCG [RD1^BCG^], *M. microti* [RD1^mic^], and *M. pinnipedii* [RD1^seal^], genes Rv1510 [RD4], Rv1970 [RD7], Rv3877/8 [RD1], and Rv3120 [RD12], insertion element IS*1561*, the 16S RNA gene, and gene Rv0577 [*cfp32*]), including the newly characterized mongoose-specific deletion in RD1 (RD1^mon^), in order to demonstrate the presence of *M. mungi* DNA in infected mongooses and investigate pathogen invasion and exposure mechanisms. *M. mungi* DNA was identified in 29% of nasal planum samples (*n* = 52), 56% of nasal rinses and swabs (*n* = 9), 53% of oral swabs (*n* = 19), 22% of urine samples (*n* = 23), 33% of anal gland tissue (*n* = 18), and 39% of anal gland secretions (*n* = 44). The occurrence of extremely low cycle threshold values obtained with qPCR in anal gland and nasal planum samples indicates that high levels of *M. mungi* can be found in these tissue types. Histological data were consistent with these results, suggesting that pathogen invasion occurs through breaks in the nasal planum and/or skin of the mongoose host, which are in frequent contact with anal gland secretions and urine during olfactory communication behavior. Lesions in the lung, when present, occurred only with disseminated disease. No environmental sources of *M. mungi* DNA could be found. We report primary environmental transmission of an MTC pathogen that occurs in association with social communication behavior.

## IMPORTANCE

Organisms causing infectious disease evolve modes of transmission that exploit environmental and host conditions favoring pathogen spread and persistence. We report a novel mode of environmental infectious disease transmission that occurs in association with olfactory secretions (e.g., urine and anal gland secretions), allowing pathogen exposure to occur within and between social groups through intricate social communication behaviors of the banded mongoose host. The presence of *M. mungi* in these environmentally deposited secretions would effectively circumvent natural social barriers (e.g., territoriality), facilitating between-group pathogen transmission in the absence of direct physical contact, a rare occurrence in this highly territorial species. This work identifies an important potential mechanism of pathogen transmission of epidemiological significance in social species. We also provide evidence of a novel mechanism of pathogen transmission for the MTC complex, where pathogen movement in the environment and host exposure dynamics are driven by social behavior.

## INTRODUCTION

The globally important tuberculosis (TB) pathogens of the *Mycobacterium tuberculosis* complex (MTC) infect a wide range of wild and domestic animals, as well as humans, presenting a critical threat to both public and animal health (1). Pathogen transmission occurs largely through aerosol and/or oral exposure (reviewed in references 1 and 2) and can occur through direct contact or indirectly through an environmental pathway, the latter of which is a poorly understood aspect of TB epidemiology.

Increasing effort has been directed, however, at understanding how environmental transmission might contribute to MTC infection dynamics. This is particularly true for pathogens such as *M. bovis*, where cross-species transmission at the wildlife-livestock interface presents continued disease control challenges (3). Studies have documented the potential for viable MTC organisms to persist in the environment (*M. bovis*), in some instances for extended periods of time (4–6). In Michigan (United States), where there is supplemental feeding of white-tailed deer (*Odocoileus virginianus*), evidence suggests that contamination of feed via infected deer in these systems contributes to TB disease occurrence in cattle (4). This is consistent with experimental studies where calves exposed to feed used by *M. bovis*-infected white-tailed deer subsequently developed TB (7). TB infection dynamics among deer, pigs, and possums (*Trichosurus vulpecula*) in New Zealand may also be driven by ingestion of, or exposure to, *M. bovis*-infected carcasses found in the environment (8). In other detailed epidemiological studies, patterns of aggregation and behavior in wild boar (*Sus scrofa*) and red deer (*Cervus elaphus*) in Spain raise concerns that *M. bovis* environmental contamination may be contributing to observed infection dynamics between these species (9). In these systems, *M. bovis* DNA has been detected in the environment at water aggregation points, with the occurrence correlated with the size of the water hole and presence of cachectic animals utilizing the resource (10).

In host-pathogen systems where environmental transmission pathways occur, complex interacting factors will influence pathogen transmission, including host susceptibility, environmental pathogen persistence, infectiousness, and mechanism of host exposure (11), elements still largely unknown for many host species and MTC organisms. There is an urgent need to better understand the mechanisms and processes that influence MTC environmental transmission and persistence potential and the resultant disease control implications.

An emerging MTC pathogen, *M. mungi*, was identified in wild banded mongooses (*Mungos mungo*) in Northern Botswana (12, 13). This novel pathogen causes significant mortality in mongooses, threatening the persistence of smaller troops or groups. The organism was confirmed as a member of the MTC with the identification of the MPB70 target, IS*6110* element, and 16S rRNA genes (12, 14, 15). Sequencing of the *gyrB* gene (encoding gyrase B) identified single-nucleotide polymorphisms (SNPs) of the *M. tuberculosis* complex member-specific sequence that placed *M. mungi* in the lineage 6 wildlife-associated group, suggesting that these organisms share a recent common ancestor (for full details, see references 12, 16, 17, and 18). The transmission pathway for this emerging MTC pathogen was previously unknown. Here, we report the discovery of a novel environmental mechanism of MTC pathogen exposure and transmission that occurs through olfactory behavior in association with anal gland secretions and urine used in scent marking in the group-living banded mongoose.

## RESULTS

### Histological presentation of *M. mungi* infection in banded mongooses.

**FIG 1:**
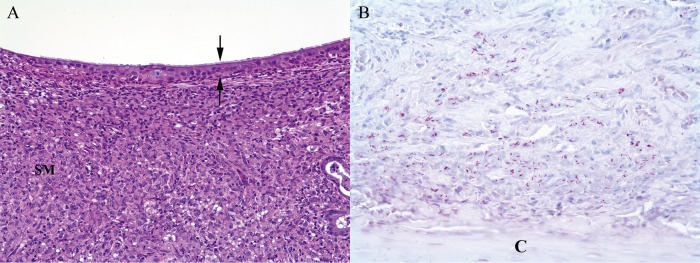
Nasal turbinate from *Mycobacterium mungi*-infected banded mongoose. (A) Submucosa (SM) is markedly expanded by infiltrates of macrophages, with abundant foamy eosinophilic cytoplasm. Lesser numbers of lymphocytes are also present. Expanded submucosa is covered by intact, ciliated nasal mucosal epithelium (between arrows) (HE staining; magnification, ×10). (B) Submucosa contains numerous magenta acid-fast bacilli. (C) Cartilage (Zeihl-Neelsen staining; magnification, ×40).

**FIG 2:**
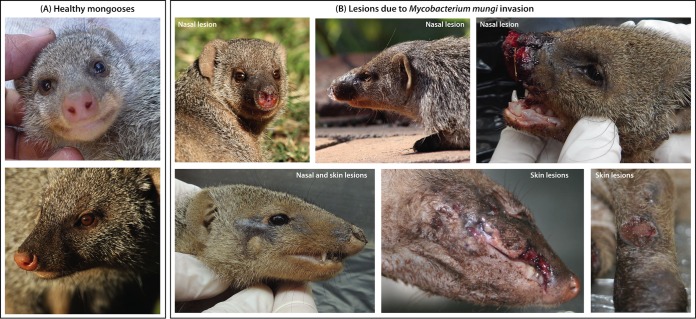
*Mycobacterium mungi* invasion occurs through injuries to the nasal planum and/or skin of banded mongooses. (A) Healthy adult mongooses. (B) Adult mongooses with advanced tuberculosis disease: tuberculosis lesions are found in the hairless parts of the mongoose nose (nasal planum and cavity), with granulomatous inflammation, erosion, and ulcerations, as well as distortion of the nasal cavity. *M. mungi* also appears to invade the mongoose host through skin lesions, often sites of previous injury. Pulmonary infection has only been detected in advanced (e.g., disseminated) disease.

We necropsied 155 mongooses from the study area from July 2000 to June 2015. Seventy-nine of these individuals were examined histologically. In *M. mungi*-infected mongooses, tuberculous lesions were found in various organs of the respiratory, gastrointestinal, lymphatic, urinary, and reproductive systems. Interestingly, lesions consistent with tuberculosis were found in 57% of nasal cavities examined (*n* = 35) and in 35% of cases where the skin of the nasal planum was examined (*n* = 34). In the nasal cavity, granulomatous infiltrates expanded the turbinate submucosa to various degrees, sometimes associated with mucosal erosions, ulcerations, and distortion of the nasal turbinates (Fig. 1A). Occasionally, granulomatous infiltrates extended into the hard palette. Ziehl-Neelsen (ZN) staining revealed numbers of intralesional acid-fast bacilli; in some cases, a myriad of acid-fast bacilli accompanied extensive granulomatous infiltration (Fig. 1B). Externally, the nasal pathology is distinctive and has not been associated with any other condition (Fig. 2).

**FIG 3:**
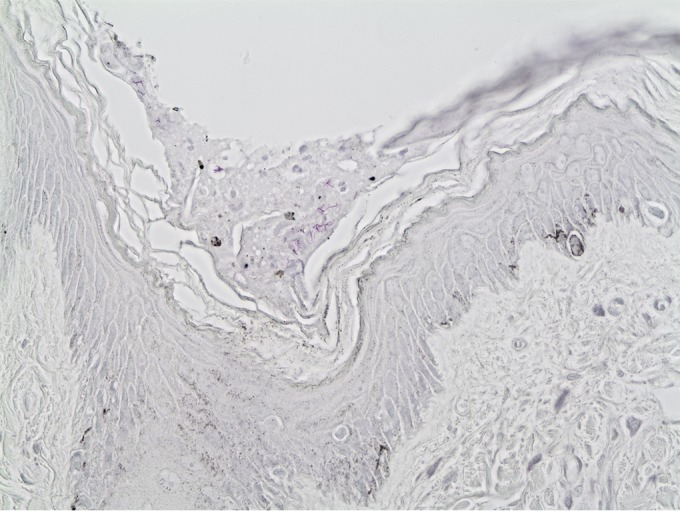
Skin sample from *Mycobacterium mungi*-infected banded mongoose. Note magenta acid-fast bacilli in debris and desquamated epithelial cells on the surface of intact epidermis. Zeihl-Neelsen staining; magnification, ×40.

Infiltrates of large numbers of macrophages and lesser numbers of lymphocytes also greatly expanded the dermis of the nasal planum. The overlying epidermis was sometimes ulcerated, with fibrin and low to moderate numbers of neutrophils associated with the ulcerated areas. In many cases, the epidermis was intact. Acid-fast bacteria were present in numbers among dermal infiltrates. Acid-fast bacilli were also seen in areas of intact epidermis and in small numbers among desquamated cells and debris on the epidermal surface, most commonly in small crevices or folds (Fig. 3).

**FIG 4:**
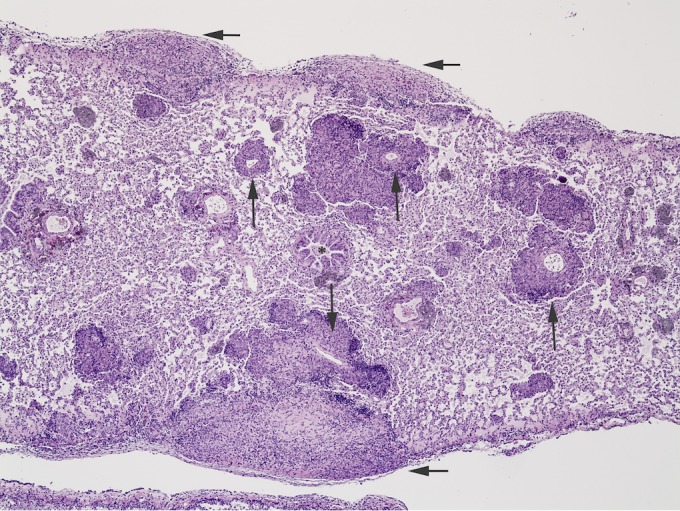
Lung tissue from *Mycobacterium mungi*-infected banded mongoose. Lung lesions are characterized by multifocal granulomas oriented around vasculature (long arrows) rather than airways (asterisk). Subpleural lesions expand the subpleural space and extend into the parenchyma (short arrows). HE staining; magnification, ×10.

Lung lesions were identified in 67% of infected mongooses examined histologically (*n* = 33), macroscopically, and microscopically; pulmonary granulomas were seen only in advanced (i.e., disseminated) cases of disease. Microscopically, pulmonary lesions consisted of variably sized nodular infiltrates of macrophages, with lesser numbers of lymphocytes, plasma cells, and multinucleated giant cells. Central caseous necrosis was variably present, and perilesional fibrosis was minimal. Dystrophic mineralization within the necrotic caseum was not seen. Intralesional intracellular acid-fast bacilli were present in increasing numbers as disease became more advanced. Microscopically, the TB pneumonia was identified as hematogenous rather than bronchogenous (i.e., by inhalation), characterized by primary association of granulomas with vasculature and, less frequently, bronchi and bronchioles (Fig. 4).

### Culture and isolation of *M. mungi.*

Initial attempts to characterize *M. mungi* involved the culture and isolation of the organism. However, *in vitro* growth of *M. mungi* was not achieved despite multiple attempts using a wide variety of tissues with visible tuberculous lesions, different incubation temperatures (28 ± 2°C and 37 ± 2°C), several different media and supplements, and different concentrations of sodium hydroxide (NaOH) during decontamination. Occasionally, atypical mycobacteria were recovered in media that were also MTC probe positive. The only colonies that were successfully recovered from solid media were *M. intracellulare*, despite a 16-week incubation of all solid media. However, *M. Mungi*-specific spoligotype and mycobacterial interspersed repetitive unit-variable number of tandem repeat (MIRU-VNTR) results were sporadically obtained when performed on DNA extracted from liquid medium cultures if DNA probes were signal positive.

### Spoligotype, MIRU-VNTR, and RD1 analyses.

**FIG 5:**
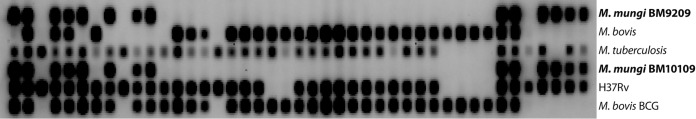
Spoligotype analysis of the emerging pathogen *Mycobacterium mungi* and representative *M. tuberculosis* complex organisms. The unique spoligotype of *M. mungi* (672600000000671) is identified in tissues of infected banded mongooses BM10109 and BM9209; MTC typing panel results are also shown in Fig. 6, and external lesions of *M. mungi* infection in mongoose BM10109 are shown in Fig. 2 (bottom right, see facial and leg lesions). Clinical diagnostic cases are included for comparison: the *M. bovis* isolate (264073777777600) was recovered from a cow, and the *M. tuberculosis* isolate (777777777760751) from a nonhuman primate. Strains H36Rv and BCG are used as controls.

**FIG 6:**
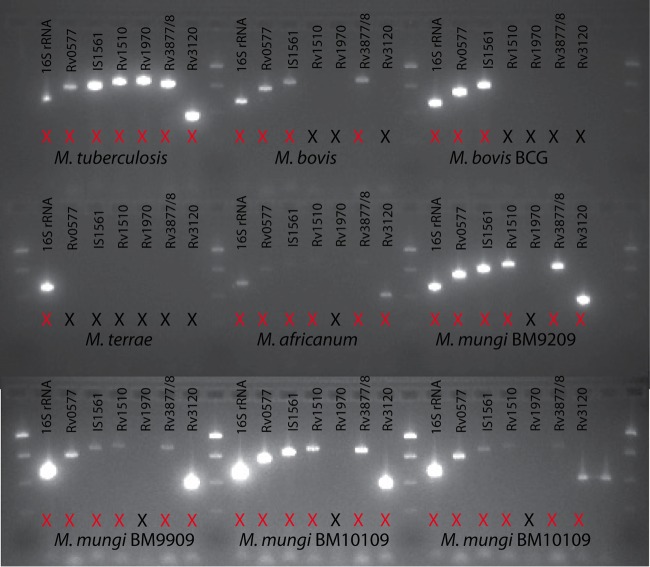
*Mycobacterium mungi* evaluation with an *M. tuberculosis* complex (MTC) typing panel. *M. mungi* can be distinguished from other MTC organisms by the presence or absence of PCR amplicons. *M. mungi* and *M. africanum* present with a similar pattern on this panel, as would the other lineage 6 members, dassie bacillus and *M. suricattae*. However, *M. mungi* can be differentiated from these MTC members by the presence of a unique deletion in region of difference 1 (RD1^mon^; see Table 2).

**FIG 7:**
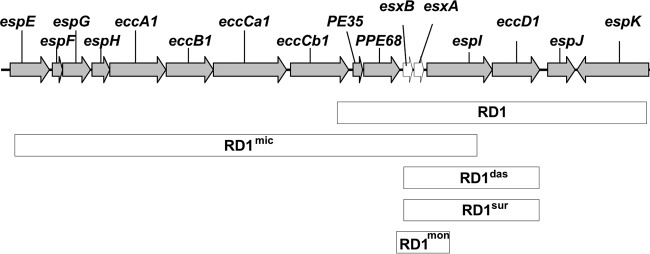
Schematic of region of difference 1 (RD1) in *Mycobacterium mungi* and other organisms.

All *M. mungi*-infected samples that were successfully spoligotyped had a unique spoligotype (SB1960) (Fig. 5) (*n* = 10 mongooses in this study and previously [12]), according to both the SpolDB4 (19) and the *M. bovis*-specific spoligotype databases (http://www.Mbovis.org). This unique *M. mungi* spoligotype has been identified in infected mongooses over several years (2000 to 2009) and across troops (*n* = 6 troops). The full set of 24 MIRU-VNTR loci (20) also identified unique patterns specific for *M. mungi* compared to the MIRU-VNTR patterns of other MTC organisms in the international database (http://www.miru-vntrplus.org) (12). In this study, we were able to obtain complete 24-locus MIRU-VNTR (MIRU-VNTR 24) patterns in three animals (*n* = 4 samples) collected in 2009, 2011, and 2013, and partial profiles in an additional 11 animals (*n* = 19 samples; see Data Set S1 in the supplemental material). The dominant pattern was 235324243821 33316354323b, with locus VNTR 2401 varying between 4 and 5. In addition to a distinctive spoligotype and MIRU-VNTR pattern, an MTC-specific multiplex PCR can be used to differentiate *M. mungi* from other members of the MTC, with the exception of *M. africanum* (Fig. 6; Table 1) (12, 16, 17, 21, 22)*. M. mungi* is distinguished from *M. africanum* by a positive result for the molecular markers region of difference 1 (RD1) from *M. pinnipedii* (RD1^seal^) and RD1 with a mongoose-specific deletion (RD1^mon^) (Table 1). The RD1^mon^ deletion provides the specific molecular marker for *M. mungi* that differentiates it from all other members of the MTC. This deletion is smaller than that found in dassie bacillus and *M.* *suricattae* (RD1^das^/RD1^sur^) (Fig. 7). From sequence data, the 3′ end of the RD1^mon^ deletion is similar to the RD1^das^ deletion, but the 5′ end of the RD1^mon^ deletion is located further upstream of the RD1^das^ deletion (GenBank accession number 1910160). PPE68 (Rv3873), the gene immediately upstream from *esxA* and *esxB*, is present in dassie bacillus and *M. suricattae* but is missing, in part, in *M. mungi*.

**TABLE 1:** Assessment of genomic regions of difference in *Mycobacterium tuberculosis* complex members^a^

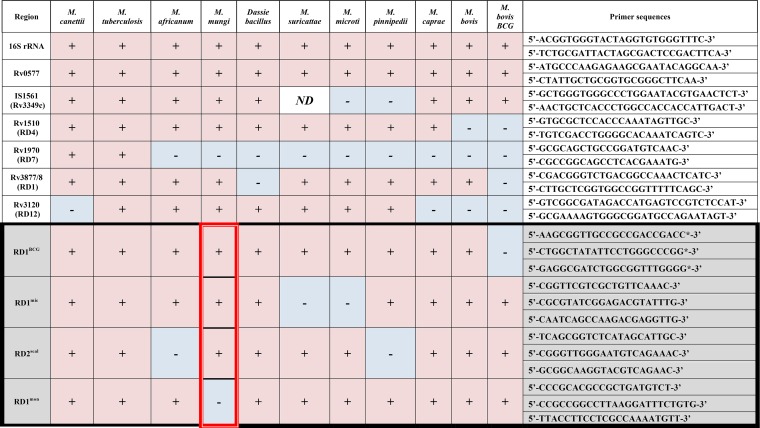	

a Samples were screened for the presence or absence of these RDs (single-nucleotide polymorphisms [SNPs] or deletions) using PCR-based typing for the specific identification of *Mycobacterium mungi* (12, 18, 21). The full sample set was then screened with a subset of these molecular markers (black box: RD1^BCG^, RD1^mic^, RD1^seal^, and RD1^mon^). These molecular markers allow the identification of any *M. tuberculosis* complex (MTC) organisms circulating in the population, as well as the presence of closely related members of the wildlife-associated lineage 6. A positive result on RD1^mon^ distinguishes *M. mungi* (red box) from all other MTC organisms. Identifications are based upon the presence of (+) or a failure to amplify (−) a PCR fragment of the expected size. RDs, regions of difference; BCG, bacille Calmette-Guérin (*M. bovis* BCG); *ND*, no data.

### Detection of *M. mungi* DNA in mongoose tissue and secretions.

As direct culture of *M. mungi* has been unsuccessful to date, we used specific molecular markers to identify the presence of DNA from any MTC organism and differentiate between lineage 6 organisms (*M. africanum*, dassie bacillus, and *M. suricattae*), as well as definitively identify *M. mungi* (Table 1). *M. mungi* DNA was identified in the anal glands and anal gland secretions in a large number of individuals (33% and 39%, respectively). Positive samples were only detected in ill mongooses, where the health status was known (Table 2). There were no differences in pathogen prevalence between anal gland tissue and secretions (*P* = 0.694, χ^2^ = 0.153). *M. mungi* was also identified in urine samples (22%), with 60% coming from clinically ill individuals. Respiratory secretions were positive for *M. mungi* DNA, consistent with histological findings of tuberculosis lesions and pathogen DNA being found in the hairless part of the mongoose nose (Table 2).

**TABLE 2:** Assessment of *Mycobacterium mungi* infection in banded mongooses

Sample type (yr[s] of sample collection)	Results for *M. mungi* detection by^a^:				
	PCR^b^			Histopathology	
	% positive	No. of samples tested	% of positive samples in agreement with necropsy result (no. positive by necropsy)	% positive	No. of samples tested
Anal glands (2009–2015)	33	18	100	—^c^	
Anal gland secretions (2009–2015)	39	44	100 (5)	—	
Oral swabs (2009–2015)	53	19	100 (3)	—	
Nasal swabs (2009–2015)	50	4	100 (1)	—	
Nasal rinses (2015)	60	5	Status unknown		
Urine (2011–2015)	22	23	60	NA^d^	
Nasal planum (2000–2015)	29	52	69	35	34
Skin lesions (2003–2015)	100	7	100 (5)	56	9
Scrotum (2015)	100	2	100	—	
Testicular lesions (2000–2008)	—			50	12
Lung (all disseminated disease) (2000–2012)	—			67	33
Bladder (2009–2015)	43	23	70	0	12
Kidney (2000–2012)	—			24	34
Feces (2013–2014)	0	113		NA	
Other species feces (see text) (2013)	0	121		NA	
Human feces in the environment (2012–2013)	0	12		NA	
Soil from infected mongoose home ranges (2011 and 2014)	0	172		NA	

a The prevalence of positive tissue samples is presented by assessment type and, of those positive, the percentage that came from individuals determined to be positive for *M. mungi* infection at necropsy.

b In PCR, samples were considered positive if primer sets for RD1^BCG^, RD1^mic^, RD1^seal^, and RD1^mon^ amplified a PCR fragment of the expected size.

c —, samples were not evaluated using the indicated diagnostic technique.

d NA, Technique not used with this sample type.

*M. mungi*-specific DNA was detected in the nasal planum (29%, *n* = 52) and skin lesions (100%, *n* = 7) (Table 2). Among mongooses positive for *M. mungi* DNA in the nasal planum, 69% of these samples were collected from clinically ill individuals, where status could be determined. *M. mungi* DNA was also identified in the scrotal sac (only two samples, both diseased) and testicles (50%, *n* = 12). In these diseased animals with positive scrotal sacs, infection in the nasal planum tissue could not be identified.

### Quantitative PCR assessment of *M. mungi* DNA in mongoose tissues and secretions.

Eighty-six tissue and secretion samples from 43 animals were tested with the National Veterinary Services Laboratories (NVSL) qPCR assay for the IS*6110-2* insertion region (23). A total of 52 tissues were positive, 27 negative, and seven inhibited (*n* = 86) (Table 3). The average cycle threshold (*C_T_*) values were lowest in the lung (26.93), liver (27.38), and nose (28.49; range, 23.76 to 35.6) and highest in the anal gland (31.93; range, 22.22 to 35.48) and anal gland secretions (35.45). The *C_T_* values found in *M. bovis*-infected cattle tissues detected at NVSL in 2015 had an average of 28.5 and a range of 22.28 to 35.53. The occurrence of extremely low *C_T_* values in anal gland (e.g., 22.22) and nasal planum (e.g., 23.76) samples indicates that the bacterial burden can be high in these tissue types.

**TABLE 3:** Quantitative PCR results from banded mongoose tissue samples submitted to the National Veterinary Services Laboratories

Sample type	No. of samples tested	No. with valid results	*C_T_* values (range)	% positive	Avg no. of *C_T_* positive samples (SD)
Anal gland	8	6	22.22–35.48	83	31.93 (5.55)
Anal gland secretions	19	18	35.35–38.68	17	36.53 (1.87)
Bladder	9	9	30.22–34.17	33	32.03 (1.99)
Liver	9^a^	6	19.25–35.5	100	27.38 (6.67)
Lung	10	10	18.4–34.53	90	26.93 (5.76)
Nose/nasal planum	14^a^	14	23.76–35.6	71	28.46 (3.87)
Skin lesion	3	3	22.77–36.48	100	30.17 (6.92)
Spleen	10	9	25.81–35.42	100	31.16 (3.56)
Kidney	2	2	23.52–35.07	100	29.30 (8.17)
Other^b^	2	2		50	20.81
Total	86	79	18.4–38.68	66	29.53 (5.23)

a The number of samples tested includes a duplicate from a single animal.

b Other samples included a foot lesion (negative) and exudate/pus.

### Assessment of *M. mungi* DNA in other potential sources of environmental exposure.

**FIG 8:**
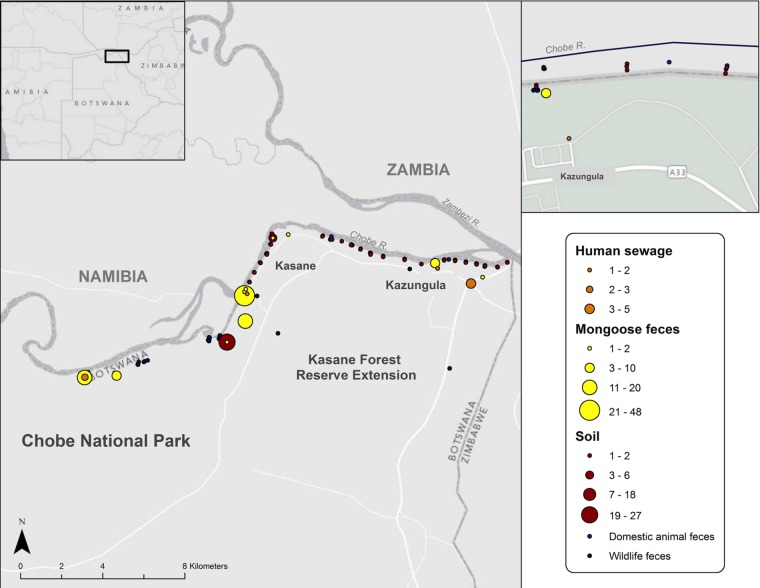
Environmental sampling sites in the home range of infected banded mongooses. The study site is within the black box on the map of Botswana (inset).

We examined other possible sources of *M. mungi* exposure, including soil (within and around dens), sewage, and mongoose feces (Fig. 8; Table 2). We also conducted a stratified survey through multiple home ranges of infected banded mongoose groups, collecting feces from various wild and domestic animals that were present in the transect and fit the selection criteria. Samples comprised 16 different species (*n* = 172 total), including African elephant (*Loxodonta africana*, *n* = 35), impala (*Aepyceros melampus*, *n* = 20), warthog (*Phacochoerus africanus*, *n* = 19), hippopotamus (*Hippopotamus amphibious*, *n* = 9), waterbuck (*Kobus ellipsiprymnus*, *n* = 6), domestic cow (*n* = 6), chacma baboon (*n* = 6), Cape buffalo (*Syncerus caffer*, *n* = 5), Chobe bushbuck (*Tragelaphus scriptus* roualeyni, *n* = 5), Southern African porcupine (*Hystrix africaeaustralis*, *n* = 4), bat-eared fox (*Otocyon megalotis*, *n* = 1), African civet (*Civettictis civetta*, *n* = 1), helmeted guinea fowl (*Numida meleagris*, *n* = 1), greater kudu (*n* = 1), lion (*Panthera leo*, *n* = 1), and vervet monkey (*Chlorocebus pygerythrus*, *n* = 1). We did not, however, identify the presence of *M. mungi* DNA from any of these sample types. Despite two decades of necropsy surveillance of wildlife and domestic animals in the Chobe region by the lead author, evidence of TB has not been identified in any species other than the banded mongoose (*n* = 46 mammalian species and *n* = 310 necropsies where records were maintained, representing a subset of all cases necropsied, some of which were histologically evaluated, from 1995 to 2015).

## DISCUSSION

This study identifies the occurrence of a novel mechanism of MTC pathogen transmission that involves environmental transmission pathways, identifying important implications for MTC pathogen transmission risk at the wildlife-environment-domestic animal interface. Macro- and microhistological and molecular genetic assessments identify that this organism is environmentally transmitted, primarily invading the mongoose host through the nasal planum and cavity and through the skin (Fig. 1 and 3), with lesions in the lung found only in advanced (e.g., disseminated) disease (12, 17). Despite extensive environmental sampling, we could only identify the presence of *M. mungi* DNA in banded mongoose tissues and secretions. The *C_T_* values detected in nose and anal gland tissue from mongooses were comparable to the *C_T_* values found in tuberculous tissue from the lung and lymph nodes of other host species infected with *M. bovis* or *M. tuberculosis*, suggesting similar tissue colonization of *M. mungi* as with other MTC pathogens. It is important to note this is the first MTC organism identified that is unable to grow *in vitro* using currently known methods. This was further supported by our inability to obtain complete MIRU-VNTR 24 sets from liquid culture. However, we successfully obtained them directly from tissue if *C_T_* values were below 21.

Where infection status could be determined, *M. mungi* DNA was detected only in the respiratory and anal gland tissues and in secretions of clinically ill mongooses, suggesting that pathogen shedding may be less frequent in mongooses with inapparent infection (the duration of which is uncertain). However, positive urine samples were collected from apparently healthy animals. While this may have important implications for the epidemiological dynamics of the system, further sampling will be needed to confirm these patterns.

While the lesion pattern and mechanism of transmission of *M. mungi* are distinct from those of other members of the MTC, natural infection and lesion formation in the skin, including occasional facial lesions, have been reported in other wildlife species infected with nontuberculous mycobacteria, including red squirrels (*Sciurus vulgaris*) infected with *M. lepromatosis* (24) and common ringtail (*Pseudocheirus peregrinus*), mountain brushtail (*Trichosurus cunningham*), and common brushtail (*Trichosurus vulpecula*) possums infected with *M. ulcerans* (25).

*M. mungi* has a unique deletion in the RD1 region (RD1^mon^), a genomic locus known to be involved in virulence and prone to deletions in other members of the MTC, such as *M. microti* (RD1^mic^), *M. suricattae*, dassie bacillus (RD1^das^), and *M. bovis* BCG (RD1^BCG^) (16, 26–28). As reported previously, the RD1^mon^ deletion has different deletion junctions than the RD1^das^ deletion (12). The RD1^mon^ deletion is relatively small (1,610 bp) compared to those of RD1^das^/RD1^sur^ (4,132 bp), RD1^BCG^ (9,456 bp), and RD1^mic^ (14,120 bp) (Fig. 7). RD1^mon^ may represent a minimal deletion of the RD1 region and shows that deletion of *esxB* and *esxA* is common to RD1 deletions from a range of MTC organisms. Characterization of this region indicates that PPE68 (Rv3873), the gene immediately upstream from *esxB* and *esxA* and present in dassie bacillus and *M. suricattae*, is missing in part in *M. mungi*. PPE68 is expressed in an operon with PE35. Both genes have been implicated in the interaction between the anti-inflammatory cytokine interleukin 10 (IL-10) and the chemokine monocyte chemoattractant protein 1 (MCP-1) in human macrophages, influencing the establishment and persistence of infection. Recent work suggests that the PE35-PPE68 interaction may modulate granuloma formation, a key element for host control of mycobacterial infections (29). This may have important implications for the immunopathology of *M. mungi* in the mongoose host.

### Pathogen transmission and olfactory behavior.

**FIG 9:**
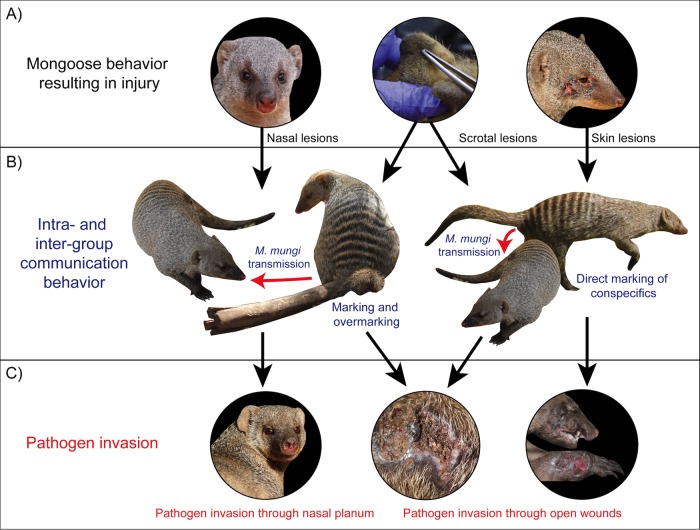
*Mycobacterium mungi* can infect the banded mongoose host through anal gland secretions and urine used in social communication behavior. (A) Banded mongooses can be injured through behavioral interactions with conspecifics, other vertebrate and invertebrate organisms, as well as injuries from the physical environment. (B) Contact with infected anal gland secretions through communication behavior allows the pathogen to invade the mongoose host through these injuries and pathogen transmission to occur between and within social groups, overcoming social barriers to transmission. (C) Pathogen invasion results in tuberculosis disease that causes high levels of mortality among banded mongooses, threatening smaller groups with extirpation (second and third photographs, mongoose BM10109; see Fig. 5 for spoligotype results and Fig. 6 for MTC typing panel results).

As with other mammalian species, banded mongooses use anal gland secretions and urine to provide critical olfactory messages to conspecifics within and between social groups (30–33). The presence of *M. mungi* in these olfactory secretions effectively allows the pathogen to hijack mongoose communication pathways. As signals, infectious secretions can both attract and expose mongooses to *M. mungi* infection within and between social groups. Additionally, with a hydrophobic cell envelope (34), *M. mungi* would be ideally adapted to the high-lipid environment of the anal gland (31). Within this lipid environment, *M. mungi* might have some level of protection from the host’s immune system and, potentially, from desiccation once deposited in the environment or on other mongooses. *M. mungi* could then be transmitted through potentially nonrandom social behaviors, such as (i) scent inspection (i.e., sniffing and contact with the nasal planum and passages), (ii) autogrooming or allogrooming in areas scent marked by infectious anal gland secretions, (iii) scent marking directly where injuries occur (i.e., through skin lesions), and (iv) overmarking on infectious anal gland secretions and contaminating injuries on the ventral/caudal aspect of the mongoose (e.g., the scrotum) (Fig. 9). This represents a novel mechanism of pathogen exposure (e.g., anal gland secretions used in scent marking) and pathogen invasion (e.g., anal gland scent investigation, marking injuries, and overmarking) (Fig. 9).

### Banded mongooses and *M. mungi* transmission: pathogen evolution and ecology.

The manner in which *M. mungi* invades, and is transmitted between mongoose hosts, spurs intriguing questions regarding TB ecology and the potential for evolution of transmission strategies that might have implications for other environmentally transmitted pathogens. Transmission of MTC pathogens typically requires close and prolonged physical contact between individuals, with respiratory (aerosol) transmission being an important mode of pathogen spread in TB maintenance hosts (e.g., humans, badgers, buffalo, and meerkats) (35–37). Transmission can also occur through oral exposure or percutaneous mechanisms (biting conspecifics) (35, 37). As a highly social and fossorial species, banded mongooses live in close and prolonged physical contact with members of their social group, particularly when the troop dens in small and enclosed environments. These life history attributes would appear to favor aerosol transmission of *M. mungi*, yet this is not the primary transmission mode of this pathogen in banded mongooses. The answer may be related to aspects of banded mongoose behavioral ecology. This species differs importantly from other TB maintenance hosts in that they are communal breeders, have a low-skew reproductive strategy (reproduction is distributed more equally among group members) (38), and exhibit a low level of extragroup mating and a lack of dispersal between groups (39). In a study conducted in Uganda from 1994 to 2001 where group history was known, no unrelated individuals emigrated into an established group during the entire study period, and females that reproduced did not have access to unrelated males (40). Additionally, in our study population, TB appears to influence dispersal directly, where clinically diseased and/or injured mongooses (injury leading significantly to TB disease) are less likely to disperse than clinically healthy conspecifics (41). While it is uncertain whether the evolution of pathogen transmission strategies in *M. mungi* has been responsive to these life history attributes, it is clear that transmission through scent marking behavior circumvents important social barriers that might normally impede pathogen transmission and spread across mongoose social groups and the population. Evolution of pathogen transmission potential has been seen in other host-pathogen systems in the MTC. For example, recent work suggests that mutations in the PhoPR regulator system have influenced virulence and transmission potential in humans among animal-adapted strains of the MTC, such as *M. bovis* and lineage 6 member *M. africanum*, explaining limited human-to-human transmission after a spillover event (42).

For the banded mongoose, infection with *M. mungi* appears to threaten group persistence of smaller troops, with larger troops remaining generally unaffected. The long-term conservation impacts are uncertain, but infection appears to be more important in mongoose populations experiencing other sources of mortality, such as car strikes and dog attacks, that depress group size.

**FIG 10:**
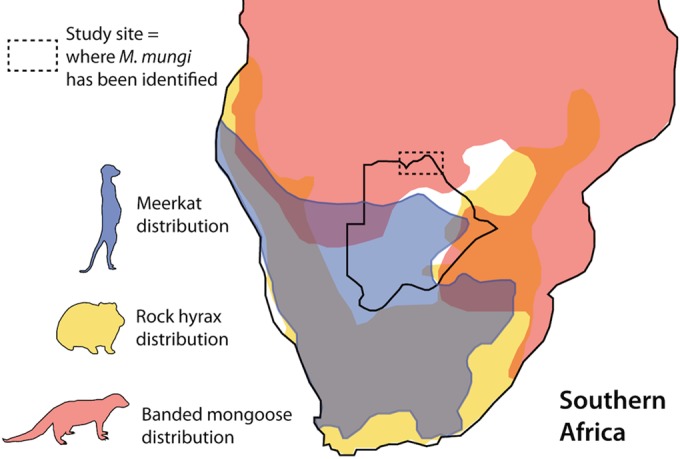
Range overlap for known reservoir hosts of lineage 6 *Mycobacterium tuberculosis* complex (MTC) organisms most closely related to *M. mungi*. The only known populations of *M. mungi*-infected banded mongoose occur in Northern Botswana and northwest Zimbabwe. The banded mongoose range distribution overlaps areas with other MTC lineage 6 reservoir hosts—the rock hyrax, *Procavia capensis* (dassie bacillus), and meerkat, *Suricata suricatta* (*Mycobacterium suricattae*). In areas where *M. mungi*-infected populations of banded mongoose have been identified (dashed line), no range overlap with these species or any other known wildlife or domestic animal reservoirs of tuberculosis occurs. Distribution data were obtained from references 58 to 60).

The data suggest that the banded mongoose is likely the definitive host and reservoir for *M. mungi*, with pathogen transmission arising from conspecifics rather than spillover from an environmental or mammalian host reservoir. The only known population of *M. mungi*-infected banded mongooses occurs in Northern Botswana and northwest Zimbabwe (Fig. 10). No range overlap is identified between this population of mongooses and host species infected with closely related MTC pathogens: the rock hyrax (*Procavia capensis*, infected by dassie bacillus) and meerkat (*Suricata suricatta*, infected by *M. suricattae*) (Fig. 10). Interestingly, of the remaining lineage 6 members, *M. africanum* and the newly discovered chimp bacillus have only been found in West Africa (17).

### Patterns of MTC pathogen transmission in other species.

In addition to aerosol transmission, *M. bovis* in the badger is believed to be transmitted through bites from individuals who have pulmonary disease, where contamination of the saliva results in bite injection (43). Similar clinical findings and assumptions have been made for meerkats infected with the MTC pathogen *M. suricattae* (44). These host species share many behavioral characteristics with the mongoose host. Both species are social and territorial and engage in complex anal gland scent marking behavior (45). Our findings suggest that pathogen transmission through scent marking should be investigated in these and other social species, particularly where pathogen transmission and infectious disease dynamics may be incompletely understood.

### Epidemiological significance: environmental transmission.

Theoretical studies find that infectious disease outbreaks involving environmental reservoirs will produce epidemic or endemic disease depending on (i) the minimum fraction of infected individuals, (ii) the minimum size of fluctuation of in-reservoir pathogens (including the nature of pathogen decay), and (iii) the shedding rate of infected individuals, also termed the pathogen enhancement ratio (46). Compartmental modeling approaches (iSIR) that incorporate in-reservoir dynamics have been applied to such systems, but important assumptions are often made; for example, assumptions regarding homogeneity in reservoir contact and pathogen shedding rates among individuals (46). In this host-pathogen system, not only does transmission through anal gland secretions and urine allow exposure without direct host contact but the secretion is also used by the mongoose host as a signal designed to directly attract other mongooses for communication purposes. These elements can facilitate pathogen exposure and secondary transmission over expected values, ultimately increasing the basic reproductive rate of the pathogen (*R*_0_)—a measure of outbreak severity (47). Future work is needed to refine our understanding of these dynamics, including how heterogeneities in individual scent marking behavior and pathogen shedding drive disease transmission. These host-pathogen dynamics may have important consequences for epidemic behavior and present new challenges for computational characterization of these systems, particularly when environmental pathogen transmission can be influenced by the dynamics of social behavior.

### Conclusions.

We identify the occurrence of a novel environmental transmission pathway for the newly emerging TB pathogen *M. mungi*. This organism is transmitted through environmentally deposited olfactory secretions, a transmission dynamic that may occur in other species, warranting further investigation in those systems. The presence of *M. mungi* in olfactory secretions effectively circumvents natural social barriers (e.g., territoriality) to pathogen transmission, potentially increasing between-group pathogen transmission in the absence of direct physical contact between infected and susceptible hosts. This has critical implications for TB outbreak potential among wildlife and domestic animals, increasing transmission opportunities across the landscape. Our work identifies environmental transmission of an MTC pathogen, highlighting the array of complex interdependent factors that may influence this route of transmission and the associated outbreak dynamics.

## MATERIALS AND METHODS

### Mongoose sampling.

Banded mongoose study troops (*n* = 8) were intensively monitored from 2000 to 2004 and from 2008 to 2015, with one or two animals radio-collared in each troop. Samples were collected antemortem from animals captured in association with radio-collaring activities and postmortem from mongoose carcasses opportunistically found in our study area during this same period (hit by car, attacked by dogs, killed by wildlife management officers, etc.). We screened anal gland tissue (postmortem only), anal gland secretions, and urine samples from mongooses from these infected study troops. To minimize pain and distress during capture procedures, we used an analgesic sedative at a dose that provides anesthesia and analgesia (1 to 1.4 mg of medetomidine and the reversal agent, atipamazole, 1:1 reversal agent volume). Animals were sampled and collared in less than 20 min, with administration of the reversal agent leading to the full recovery of an individual within 5 min. Animals were able to join their respective troop in less than 40 min with minimized disturbance to the individual and group.

To collect anal gland samples from anesthetized mongooses, animals were placed in dorsal recumbency and anal glands were digitally palpated and, once located, manually expressed into a sterile tube. Antemortem, urine was collected by placing a sterilized rubber mat in close proximity to a study mongoose troop. Once an individual mongoose urinated on the mat while investigating, the sample was collected using a transfer pipette and placed into a sterile 1.5-ml tube. The mat was cleaned with 10% bleach and sterilized water in between sample collections. Nasal and oral swabs were collected antemortem from anesthetized animals, as well as during postmortem examinations. A sterile swab was gently run over nasal or oral surfaces and then placed in a sterile 1.5-ml tube. More recently, we have moved to nasal rinses, where sterile saline is flushed into the nasal cavity and collected into a sterile tube. Mongoose fecal samples were collected from six infected troops following morning latrine behavior as previously described (48). Each morning upon leaving their den, banded mongooses will defecate individually during the same time period and in the same general location (referred to as a latrine), making it possible to collect fecal samples from individual mongooses in each troop without replication during that latrine event.

All animal handling and sampling activities in this study were conducted under approval from the Virginia Tech Institutional Animal Care and Use Committee (IACUC 13-164-FIW), as well as the Botswana Ministry of Environment, Wildlife and Tourism (EWT 8/36/4 XXVI) (24).

Animals were classified as having TB if an experienced prospector identified macroscopic lesions consistent with TB at necropsy (i.e., variably sized, grayish to white, nodular lesions on any organ). The majority of these cases were later confirmed by histopathology and/or PCR (*n* = 105). Clinical signs of *M. mungi* infection include anorexia to cachexia, hunched body posture, matted fur, epiphora, sneezing, rhinorrhea, nasal enlargement, deviation of the nasal septum, drooping and/or enlarged testicles, lethargy, lagging behind the group, and fearlessness. The clinical and gross pathological presentation of *M. mungi* (early to late stage) is very distinctive and has not been associated with any other disease syndrome in the mongoose host over the last 15 years of research (12). A syndromic approach to observational health classification has been employed previously where a visible and specific clinical presentation is predictive of pathogen infection (e.g., *Mycoplasma gallisepticum* infection in house finches, *Carpodacus mexicanus* [49]).

### Environmental sample collection.

Samples from wildlife and domestic animal feces, soil, and sewage were collected along foot transects in the home range of infected banded mongoose study troops across protected and unprotected areas (Fig. 8). Environmental sampling was opportunistic and did not involve the capture or handling of any live animals or engage human subjects.

Fecal and sewage samples were collected three times in July, August, and September of 2011, as previously described (50). Briefly, 55 stratified transect points were identified, 100 m in length, perpendicular to the river, spaced at 500 m intervals along the Chobe River, which traverses the study area and the ranges of infected mongoose troops that live in the area. Sampling started at the confluence of the Chobe and Zambezi Rivers (transect 1, −17°47′39.9114″, 25°15′38.5554″) and extended 27.5 km upstream, into the Chobe National Park (transect 55, −17°49′55.4154″, 25°2′53.0874″). Using aseptic techniques, a sample was taken from the center of the fecal ball using a sterile tongue depressor and collected in a sterile 50 ml conical tube. Feces that could not be reliably identified due to disruption and poor surface type (failed spoor or foot print detection) or were older than 24 h (as determined by an experienced wildlife tracker), were excluded from the study. Complete sampling (transects 1 to 55) took approximately 5 days. Sampling was conducted three times at one month intervals during the sampling period. Soil samples were collected along the same transects from June to July of 2014. Samples were collected at the beginning, middle, and end point of each transect, at the surface and 4 inches below the surface. Samples were also collected in and around den sites.

### Histology.

During necropsies, samples of all major organs, as well as all of the lymph nodes (marked separately for identification: submandibular, prescapular, popliteal, hilar, and mesenteric), were collected and fixed in 10% buffered formalin for histological examination. All macroscopically visible lesions were also sampled. Formalin-fixed tissue sections were prepared using routine techniques and stained with hematoxylin and eosin (HE). Ziehl-Neelsen staining was used to visualize acid-fast bacilli.

### Culture and isolation.

Tissue samples, including spleen, liver, submandibular lymph nodes, and skin from diseased animals, were homogenized in phenol red broth, decontaminated with various concentrations (0.7 to 2%) of NaOH for 7 to 10 min, and neutralized back to a pH of approximately 7 based on phenol red color indicator, using 6 N HCl (51, 52). After centrifugation at 4,600 × *g*, the sediments were inoculated into standard Bactec MGIT tubes (Becton, Dickinson, Sparks, MD) using in-house 7H9 broth and the following solid media: 7H10 medium supplemented with glycerol or pyruvate, Stonebrink medium, Lowenstein-Jensen medium, 7H11 medium supplemented with egg yolk and mycobactin J, and 7H100 medium supplemented with 10% calf serum, 5% hemolyzed blood, malachite green, and pyruvate. Additional MGIT tubes were also supplemented with hemin and NAD strips (BBL strips; Becton, Dickinson). Skin and nasal tissue sediments were incubated at both 28°C and 37°C, while internal organ tissues were incubated at 37°C. MGIT medium was incubated for 49 days, and solid medium tubes were read weekly for the first 8 weeks and then held for a final read at 16 weeks. Commercial DNA probes (Hologic, San Diego, CA) were used to identify MTC organisms in signal-positive MGIT tubes.

### MTC differential PCR, spoligotyping, and MIRU-VNTR analyses.

DNA for molecular testing was extracted from liquid medium after incubation. Briefly, 500 µl of the liquid medium was placed in Tris-EDTA and phenol-chloroform and bead disrupted, and the aqueous layer purified by ethanol precipitation. The TB species differential PCR was performed as previously published, amplifying each primer separately and then visualizing the products on an agarose gel (53). The same extracted DNA was also subjected to spoligotyping by amplifying the spacer regions using PCR and visualizing their presence/absence using the Southern blotting technique (54). The MIRU-VNTR 24 was also performed on DNA by multiplexing primers for three loci in eight PCRs and then conducting fragment analysis on an ABI 3500XL instrument (Thermo Fisher Scientific, Waltham, MA, USA) (20).

### Direct tissue qPCR.

Lesioned tissue was weighed, placed in a bead disruption tube with Tris-EDTA and DNA extraction control 670 (DEC670) (Bioline, London, United Kingdom), and heat inactivated. After bead disruption, the DNA was isolated with phenol-chloroform and purified by mixing the aqueous layer with 1.2 ml DNA binding buffer (Zymo Research, Irvine, CA, USA), loading onto a Zymo-Spin I-96 plate (deep well) (Zymo Research), rinsing with buffers, and eluting with 100 µl buffer. Five microliters of the purified DNA was used in a quantitative real-time PCR with IS*6110-2* primers (forward, 3′-ACACATCGATCCGGTTCAGC-5′; reverse, 3′-TCGTCTCGGCTAGTGCATTG-5′; and probe, 3′-TCGGTCGGAGCGGTCGGAAG-5′) (23).

### Tissue, secretions, and environmental source sample screening.

DNA from all mongoose tissues and anal gland secretions was extracted using the protocol for Gram-positive bacteria in the Qiagen DNeasy blood and tissue kit (Qiagen, Hilden, Germany), modified to include a chemical lysis pretreatment step. DNA from urine was extracted using the Norgen BioTek urine DNA isolation kit for exfoliated cells or bacteria (Norgen Biotek Corp., Ontario, Canada) with a similar pretreatment step. DNA was extracted from environmental samples using the Power fecal DNA isolation kit and the Power soil DNA isolation kit (Mo Bio Laboratories, Inc.) after the pretreatment step.

For characterization of RD1^mon^, primers were designed to target the flanking region to amplify the mongoose-specific deletion. Products were sequenced to determine the extent of the deletion and refine RD1 primer sets to be used for detection of *M. mungi* in this study.

To confirm *M. mungi*-specific DNA in a sample, extracted DNA was screened for the presence or absence of RDs (single-nucleotide polymorphisms [SNP] or deletions) using PCRs that allowed the identification of *M. mungi* DNA (Table 1, primer sets for amplification of 16sRNA, Rv0577, IS*1561*, Rv1510, RV1970, Rv3877/8, Rv3120, RD1^BCG^, RD1^mic^, RD1^seal^ [12, 18, 21], and RD1^mon^). The full sample set was then screened for a subset of these molecular markers (RD1^BCG^, RD1^mic^, RD1^seal^, and RD1^mon^), with positive or negative status determined by the presence of (+) or a failure to (−) amplify a PCR fragment of the expected size (RD1^BCG^, +146 bp/−196 bp; RD1^mic^, +195 bp/−127 bp; RD1^seal^, +293 bp/−168 bp; and RD1^mon^, +700/−less than 5,062 bp but greater than 700 bp). These specific molecular markers were selected to identify *M. mungi* and ensure the identification of any other MTC organism circulating in the population, differentiating the presence of closely related members of lineage 6 (e.g., *M. africanum*, dassie bacillus, and *M. suricattae*)*.* A positive result on RD1^mon^ distinguishes *M. mungi* from all other MTC.

PCR products from the primer sets were initially sequenced to ensure that the correct products were being amplified. *M. tuberculosis* H37rv and *M. mungi* DNA extracted from a confirmed case (mongoose BM10109) were used as positive controls. *M. mungi* infection in BM10109 was confirmed using spoligotyping (Fig. 5), MIRU-VNTR (see Data Set S1 in the supplemental material), gross pathology (Fig. 9), histology, and the MTC typing panel (Fig. 6), including the additional primer sets provided in Table 1. PCR amplifications were performed in 25 µl reaction mixtures containing 5× HotStarTaq Plus master mix (Qiagen, Hilden, Germany), 1× Q solution, and 0.5 µM forward and reverse primers. PCR amplifications were performed as described above, except that the initial activation was followed by 45 cycles at 94°C for 1 min, 62°C for 1 min, and 72°C for 1 min (21).

To ensure that our DNA extraction and PCR protocols were functional by sample type, we experimentally spiked each sample type (urine, anal gland, fecal, sewage, and soil) with extracted *M. mungi* DNA (1 µl), yielding positive PCRs consistent with the controls. We also assessed the sample types for the presence of 16S rRNA genes (a gene used to identify bacteria, in particular, mycobacteria [55], which did not require samples to be spiked) and again, identified PCR-positive samples among all sample types, confirming that our test results were accurate and not a consequence of unknown upstream PCR inhibitors.

PCR products were visualized on 2% agarose gels, providing increased ability to resolve ambiguity in amplicon size indicative of variable genomic sequences. Gels were stained with ethidium bromide and visualized on the Bio-Rad Gel Doc XR+ imager (Bio-Rad, CA, United States). For all sequencing, positive bands were excised from these gels and the DNA was extracted using the QIAquick gel extraction kit (Qiagen, Hilden, Germany). The Virginia Bioinformatics Institute (VBI) at Virginia Tech and the Albert Einstein College of Medicine provided sequencing services. Sequences were searched against the nucleotide Basic Local Alignment Search Tool (BLAST) of the GenBank database (available at http://blast.ncbi.nlm.nih.gov).

### Study limitations.

*M. mungi* DNA was definitively found in olfactory secretions and within the nose and skin of infected mongoose hosts, findings that were further confirmed from gross and histopathological examinations. MTC pathogens are not ubiquitous across a tissue type, and PCR evaluations may miss the presence of a pathogen, potentially underestimating the true number of *M. mungi*-positive samples.

Extensive chemical analysis and behavioral studies have previously been conducted on banded mongoose anal gland secretions, as well as scent marking behaviors (31, 32, 56), with findings from this and other work utilized to interpret our results. While we conclude that environmental pathogen transmission from anal gland secretions and urine are important in *M. mungi* disease transmission and presentation, it is not possible to conclude that this is the only mechanism of disease exposure and host invasion. We could not, however, identify *M. mungi* in any other environmental sources surveyed. Experimental duplication of pathogen invasion through scent marking behavior in the mongoose host would provide a “gold standard.” This experiment would not be possible, as is the case with most wildlife disease studies. Experimental manipulations that involve wild animals and infectious disease are impractical to pursue and, furthermore, extremely difficult, if not impossible to justify, particularly in Northern Botswana and northwest Zimbabwe, the only known location where this species is infected with *M. mungi*.

While we sampled the environment extensively, including den areas of mongoose troops, we cannot exclude the possibility that environmental sources of *M. mungi* still persist at some extremely low level. As previously mentioned, studies of the environmental persistence of *M. bovis* identified extended periods of survival in soil, with a survival optimum occurring at 37°C in moist soils (57). Dead cells of *M. bovis* BCG (Pasteur) did not persist more than 10 days in these environments. Lack of environmental detection within the soil in our system would be consistent with features of this region of Africa: Botswana is an arid country with soils of predominantly Kalahari sands and has a limited rainy season, largely sunny days, and temperatures that regularly exceed 37°C (100°F).

### Data set accession number.

Genetic data have been deposited in the publicly accessible GenBank sequence database under accession number KX174310.

## SUPPLEMENTAL MATERIAL

Data Set S1  Mycobacterial interspersed repetitive unit-variable number of tandem repeat (MIRU-VNTR) results for *Mycobacterium mungi*-positive samples. Download Data Set S1, XLSX file, 0.1 MB

Data Set S2  Results of quantitative PCR assessment of *Mycobacterium mungi* DNA in mongoose tissues and secretions. Download Data Set S2, XLSX file, 0.1 MB
