# Erratum for Alexander et al., Emerging Tuberculosis Pathogen Hijacks Social Communication Behavior in the Group-Living Banded Mongoose (*Mungos mungo*)

**DOI:** 10.1128/mBio.00921-16

**Published:** 2016-06-21

**Authors:** Kathleen A. Alexander, Claire E. Sanderson, Michelle H. Larsen, Suelee Robbe-Austerman, Mark C. Williams, Mitchell V. Palmer

**Affiliations:** a Department of Fish and Wildlife Conservation, Virginia Tech, Blacksburg, Virginia, USA; b CARACAL, Centre for Conservation of African Resources: Animals, Communities and Land Use, Kasane, Botswana; c Department of Medicine, Albert Einstein College of Medicine, Bronx, New York, USA; d Diagnostic Bacteriology Laboratory, National Veterinary Services Laboratories, Ames, Iowa, USA; e University of Pretoria, Onderstepoort, South Africa; f National Animal Disease Center, Bacterial Diseases of Livestock Research Unit, Ames, Iowa, USA

## ERRATUM

Volume 7, no 3, doi:10.1128/mBio.00281-16, 2016. On PDF page 5, first column, the GenBank accession number “1910160” should be updated to “KX174310.” The correct URL is http://www.ncbi.nlm.nih.gov/nuccore/KX174310.

On PDF page 11, second column, “RD1^mon^, +700/− less than 5,062 bp but greater than 700 bp)” should be updated to “RD1^mon^, +309 bp/−513 bp).”

